# Autophagy, endolysosomes and extracellular space in autoimmune and innate immune defects

**DOI:** 10.3389/fimmu.2026.1947320

**Published:** 2026-10-07

**Authors:** Juliane Schuler, Lea Tollmann, Robert Walter Körner, Birk Möller, Emil Schober, Charlotte Schöner, Maaike Kusters, Ming Lim, Tim Niehues, Heinz Jungbluth, Hormos S. Dafsari

**Affiliations:** 1 Department of Pediatrics, Faculty of Medicine and University Hospital Cologne, University of Cologne, Cologne, Germany; 2 Max-Planck-Institute for Biology of Ageing and Cologne Excellence Cluster for Ageing-Associated Diseases, Cologne, Germany; 3 Department of Immunology, Great Ormond Street Hospital for Children NHS Foundation Trust, London, United Kingdom; 4 UCL Great Ormond Street Institute of Child Health, Departement of Infection, Immunity & Inflammation, University College London, London, United Kingdom; 5 Children’s Neuroscience, Evelina London Children’s Hospital at Guy’s and St Thomas’ NHS Foundation Trust, London, United Kingdom; 6 Faculty of Life Sciences and Medicine, King’s College London, London, United Kingdom; 7 Department of Neurology, Hunan Children’s Hospital, Changsha, China; 8 Helios Klinikum Krefeld, HMU Health Medical University Campus Düsseldorf/Krefeld, Düsseldorf, Germany; 9 Randall Centre for Cell and Molecular Biophysics, Muscle Signaling Section, Faculty of Life Sciences and Medicine (FoLSM), King's College London, London, United Kingdom; 10 Center for Rare Diseases, Faculty of Medicine and University Hospital Cologne, University of Cologne, Cologne, Germany; 11 Center for Molecular Medicine Cologne (CMMC), Faculty of Medicine and University Hospital Cologne, University of Cologne, Cologne, Germany

**Keywords:** autoimmunity, autophagy, endosomes, innate immunity, neutrophil extracellular traps

## Abstract

Autophagy is increasingly recognized as a central immunological process whose roles extend far beyond intracellular recycling. By coordinating lysosomal degradation, organelle quality control, selective antimicrobial clearance, and inflammatory signal termination, autophagy acts as a decisive checkpoint at the crossroads between protective innate immunity and pathological immune persistence. Xenophagy, the selective autophagic elimination of intracellular pathogens, is a particularly important process at the interface between host defense, endosomal trafficking and immune resolution. Distinct monogenic disorders affecting *EPG5*-dependent autophagosome-lysosome fusion, *LYST*-dependent lysosomal and granule organization, or *RAB27A*-dependent lytic-granule exocytosis illustrate how different lesions in intracellular trafficking can impair microbial handling and contribute to immune dysregulation. Functioning autophagy also boosts cellular resilience through the removal of intracellular debris via exocytosis. In parallel, neutrophil extracellular traps (NETs) have emerged as potent antimicrobial structures that also aid cellular resilience but have a dual role in immunity: while NET formation can restrict pathogen dissemination, excessive or insufficiently cleared NETs expose chromatin, histones, proteases and modified autoantigens that can drive autoimmunity, thromboinflammation and tissue damage. In this review, we discuss the intricate links between xenophagy, its intracellular endolysosomal hubs, and its extracellular targets and traps as an integrated cell-biological and immunopathological network. We highlight how autophagy initiation and verified degradative flux, mitochondrial quality control, reactive oxygen species, peptidylarginine deiminase 4 (PAD4)-dependent chromatin decondensation, and endolysosomal trafficking may intersect with neutrophil effector functions in a stimulus- and model-dependent manner. Particular emphasis is placed on endosomal checkpoint proteins such as TOM1, which link ubiquitinated cargo sorting, IL-1R1/NF-κB signal termination, autophagosome maturation, and lysosomal degradation. We propose that defective endosomal and autophagic resolution mechanisms blur the boundary between efficient antimicrobial defense and chronic inflammatory auto-amplification. This concept is highly relevant for a wide range of autoimmune conditions, including systemic lupus erythematosus, rheumatoid arthritis, ANCA-associated vasculitis, autoinflammatory syndromes, immunodeficiency, and neuroinflammatory disease. Finally, we discuss therapeutic strategies aimed at restoring immune resolution, including modulation of autophagy, enhancement of NET clearance, targeting of PAD4 and inflammasome pathways as well as correction of endolysosomal trafficking defects. Understanding these intricately linked networks may open new avenues for mechanism-based treatment of autoimmune and innate immune disorders.

## 1 Introduction

Autophagy is a degradative housekeeping pathway that regulates whether potentially harmful cytoplasmic cargo is eliminated via cargo disassembly within lysosomes or exported into the extracellular space (1). Based on the mode of cargo trafficking to lysosomes, autophagy can be divided into macroautophagy, microautophagy, and chaperone-mediated autophagy (2). In canonical macroautophagy, hereafter referred to as autophagy, cytoplasmic material is sequestered within a double-membraned autophagosome and subsequently delivered to lysosomes for degradation and recycling. This sequence of events - phagophore initiation, elongation, closure, autophagosome maturation and autophagosome-lysosome fusion - maintains cellular homeostasis during periods of increased metabolic demands and allows cells to remove damaged proteins, dysfunctional organelles and invading microorganisms. The final fusion step is the basis for successful clearance and therefore particularly relevant for immune homeostasis as cargo recognition without lysosomal degradation can leave biologically active inflammatory material within the cytoplasm unless removed from the cells via exocytosis. The clinical relevance of this principle is illustrated by congenital autophagy disorders, in which impaired autophagosome-lysosome fusion is associated with neurological diseases, including neurodegeneration and seizures related to synaptic malfunction, as well as immunological manifestations such as recurrent infections, immunodeficiency, and immune dysregulation with hyperinflammation (3, 4).

Xenophagy represents the antimicrobial and immunological selectivity within the autophagic machinery. In xenophagy, intracellular pathogens or pathogen-damaged vacuoles are marked, frequently by ubiquitin, and recognized by selective autophagy receptors or adaptors such as p62/SQSTM1, NDP52, and optineurin. These adaptors bridge cargo to LC3-positive membranes and designate microbial structures for lysosomal destruction, a process similar but not identical with LC3-associated phagocytosis and other non-canonical ATG8-conjugation pathways in primary phagocytes (1). Together, these pathways form an endolysosomal defense network in which recognition, vesicular maturation, and degradative completion determine whether microbial material is neutralized or remains available for persistent innate immune sensing.

The immunological relevance of autophagy in the form of xenophagy extends beyond direct pathogen elimination. Whilst pathogen elimination is the primary function, these pathways also regulate the duration and intensity of pattern-recognition receptor signaling, restrain inflammasome activation, remove damaged mitochondria and cytosolic nucleic acids, and influence antigen handling in antigen-presenting cells. Autophagy also contributes to B-cell and T-cell biology by shaping intracellular nucleic acid trafficking, antigen presentation, lymphocyte survival and metabolic programming. In *EPG5*-related disorders such as Vici syndrome, impaired autophagosome-lysosome fusion disrupts nucleic acid trafficking and TLR9 compartmentalization, linking defective autophagic clearance to both innate and adaptive immune abnormalities (5, 6). This paradigmatic disorder thus demonstrates the crucial position of autophagy at the interface between antimicrobial defense and immune tolerance.

This interface is particularly relevant in autoimmunity, as intracellular material destined for degradation may persist as an inflammatory trigger when autophagic or xenophagic clearance is incomplete. As a result, damaged mitochondria may persist and generate reactive oxygen species, cytosolic DNA and RNA may remain accessible to innate sensors, and endosomal receptors may continue to signal because of delayed receptor degradation (7). The consequence is not simply excessive activation, but failed resolution, with inflammatory programs remaining active because the intracellular signaling pathways required for their termination are impaired.

This vicious circle model may explain why disorders of autophagy, endosomal transport, and lysosomal fusion hardly ever follow a singular and linear immune phenotype but rather combine infection susceptibility with enhanced autoimmunity and hyperinflammation. Such a model suggests complex pathomechanisms that may reflect biological reality more closely than previously assumed, and raises the question of how cells tolerate such complex stressors.

This review discusses the biological basis of intracellular resilience in relation to autophagy, endolysosomal pathways and NET formation, an extracellular effector process that may intersect with, but is not simply downstream of, intracellular trafficking and recycling. Neutrophils deploy chromatin-based antimicrobial structures enriched with granule-derived enzymes and histones as neutrophil extracellular traps (NETs). Whilst this mechanism is primarily a beneficial component of the immune response, when NET formation is excessive or NET clearance is incomplete, the same material can become a source of autoantigens and damage-associated molecular patterns.

Autophagy-related processes may affect NET formation in opposing, context-dependent ways. Efficient degradative flux and xenophagy can reduce upstream triggers by eliminating pathogens, damaged mitochondria, and inflammatory cargo, whereas early ATG-dependent membrane or metabolic responses have been reported to support lytic NET release under selected stimuli. Importantly, LC3 recruitment or autophagosome accumulation does not demonstrate completed lysosomal degradation. The autophagy-NET relationship therefore depends on stimulus, cell state, the experimental definition of NET formation, and the pathway stage measured (8, 9).

## 2 Neutrophil extracellular traps: formation, regulation, and role in immunity

Neutrophil Extracellular Traps (NETs) are web-like structures composed of a DNA backbone decorated with antimicrobial proteins (e.g., myeloperoxidase, neutrophil elastase) and histones. Neutrophils are the most abundant myeloid leukocytes. They differentiate from hematopoietic stem and progenitor cells in the bone marrow, circulate in the bloodstream, migrate to the sites of infection and transmigrate through vessels into tissues, where they act as key effectors of innate immune defense (10). Beyond classical antimicrobial mechanisms such as phagocytosis and the oxidative burst, NET release was initially described in association with a lytic neutrophil-death program. NET formation, however, should not be used synonymously with programmed cell death because rapid vital NET release can occur without plasma-membrane rupture or immediate loss of neutrophil function (9). NET formation can be triggered by bacteria, fungi, viruses, pathogen-associated molecular patterns (PAMPs), such as lipopolysaccharide (LPS), activated platelets, and cytokines. In lytic NET formation, chromatin decondenses, nuclear and granule membranes disassemble, nuclear and cytoplasmic components mix, and the plasma membrane ultimately ruptures to release NETs (9).

At least two operational modes of NET release are recognized. Classical or lytic NETosis is an irreversible death-associated process involving granule-protein translocation, extensive chromatin decondensation, nuclear-envelope disintegration, and eventual plasma-membrane permeabilization; gasdermin D can contribute to membrane pore formation in selected settings. Many lytic models are reactive-oxygen-species (ROS) dependent and develop over several hours (10). Phorbol 12-myristate 13-acetate (PMA) is commonly used to model slow, ROS-dependent lytic NET formation through protein kinase C (PKC)- and autophagy-related signaling (11). The role of NADPH in vital NETosis remains less well characterized. By contrast, vital NET formation can occur rapidly while preserving short-term chemotaxis and phagocytosis; its triggers and dependence on NADPH oxidase vary by stimulus (10).

NET formation is tightly regulated. Surface receptors such as Toll-like receptors (TLRs), TNF receptors, and complement receptors initiate activation (12). Subsequently, calcium influx from the endoplasmic reticulum (ER) and plasma membrane channels stimulates PAD4, which requires high calcium for histone citrullination, while serine proteases regulate chromatin decondensation downstream. PAD4 is not universally required for NET formation, as PAD4-independent forms have been described (13). The driving kinase pathways are highly stimulus-dependent, involving PKC, the Raf-MEK-ERK, CDK6, and the SYK-PI3K-mTORC2 pathway (13).

Evidence linking autophagy-related processes to NET formation is heterogeneous and stimulus dependent. In selected ROS-dependent lytic models, genetic disruption of ATG proteins or pharmacological manipulation of PI3K-mTOR pathways has reduced or enhanced trap release. Pharmacological tools can also affect pathways beyond autophagy, and LC3 recruitment or autophagosome accumulation does not by itself demonstrate completed autophagic flux. Autophagy initiation, LC3 lipidation, autophagosome abundance, fusion with lysosomes, and lysosomal cargo degradation should therefore be distinguished when interpreting effects on NET formation (9).

NET formation plays a dual role in immunity, serving as a critical innate defense mechanism but also, when dysregulated, as a driver of (auto)immunopathology (10). NETs mediate antimicrobial defense by physically ensnaring pathogens and exposing them to high local concentrations of bactericidal granule proteins such as neutrophil elastase and myeloperoxidase. NETs can also promote immunothrombosis, a localized coagulation response that helps capture pathogens and restrict their dissemination (10). However, excessive NET formation or insufficient NET clearance can become detrimental, as NET-derived damage-associated molecular patterns, including double-stranded DNA, histones, and citrullinated autoantigens, may stimulate antibody production and contribute to autoimmune diseases such as systemic lupus erythematosus and rheumatoid arthritis (10). Pathological NET accumulation also contributes to immunothrombotic complications, including ischemic stroke and myocardial infarction, as well as tissue damage in inflammatory lung diseases such as acute respiratory distress syndrome, chronic obstructive pulmonary disease, and severe asthma. This dual nature underscores the importance of tightly regulated NET formation and clearance for maintaining immune homeostasis (10).

## 3 The endosomal pathway and autophagy: shared machinery and crosstalk

Autophagy is a highly conserved intracellular degradation pathway that delivers cytoplasmic cargo, including protein aggregates, damaged organelles and invading microorganisms, to lysosomes for breakdown and recycling (14). Autophagy can be classified into chaperone-mediated autophagy (CMA), microautophagy, and macroautophagy. In CMA, chaperones recognize specific protein motifs and deliver these proteins to lysosomes for direct translocation into the lysosomal lumen. Microautophagy is characterized by direct engulfment of cytoplasmic cargo through invagination of the endosomal or lysosomal membrane, whereas in macroautophagy intracellular components are surrounded by a dedicated double-membraned vesicle, the autophagosome (4). Macroautophagy (hereafter referred to as ‘autophagy’) is differentiated into several tightly regulated stages, the initiation of an isolation membrane (or phagophore), the elongation and closure into a double-membrane autophagosome, and finally the fusion with lysosomes to form autolysosomes, where cargo degradation occurs (14). This process is orchestrated by autophagy core genes (Atgs), SNARE complexes, and other regulatory proteins, including the tethering factor EPG5 (ectopic P-granules autophagy protein 5) (15, 16), and terminated in a final step, autophagosome-lysosome fusion followed by lysosomal cargo degradation.

EPG5 is a key component of the autophagic fusion machinery and orchestrates the interaction between Rab7-positive autophagosome and lysosomal surface proteins such as LAMP2 and VAMP7 (15, 16). The recruitment and function of EPG5 are closely linked to the activity of WDR45, which contributes to autophagosome maturation and regulates lipid-related processes required for LC3 conjugation. WDR45 indirectly enhances vesicle tethering efficiency by supporting EPG5 localization to autophagic membranes (17). These coordinated events ensure precise membrane positioning, facilitate SNARE assembly, and ultimately promote effective autophagosome-lysosome fusion. Through this integrated role, EPG5 acts as a major determinant of fusion efficiency within the autophagic pathway (15, 16).

Autophagy primarily functions as a catabolic pathway that degrades and recycles long-lived or damaged proteins and organelles to ensure cellular survival during nutrient deprivation or other cellular stress conditions. In parallel with its catabolic functions, autophagy serves as a critical quality control mechanism that maintains cellular homeostasis (3, 4, 18). In addition to its well-established role as a degradative pathway, autophagy is increasingly recognized as a central regulator of immunity, linking intracellular clearance to innate and adaptive immune responses (19).

Four principal immunological functions of autophagy have been defined: i) direct elimination of intracellular microorganisms through xenophagy and LC3-associated phagocytosis (LAP), ii) control of inflammation, iii) regulation of adaptive immunity through antigen presentation and lymphocyte homeostasis, and iv) secretion of immune mediators (19). Xenophagy is a specialized form of selective autophagy targeting intracellular pathogens for lysosomal degradation. Pathogen-containing cargo is initially tagged by ubiquitination for autophagy receptors or adaptors such as p62, NDP52 or OPTN and others. The cargo is then engulfed by the phagophore, which matures into an autophagosome, and subsequently fuses with lysosomes for degradation by lysosomal hydrolases (20, 21). While traditionally xenophagy specifically refers to the degradation of ubiquitinated bacterial pathogens, virophagy is another form of xenophagy that targets a variety of viruses independently of their ubiquitination status (19).

LC3-associated phagocytosis (LAP) is a non-canonical, single-membrane phagosome-maturation pathway that follows uptake of extracellular material. Unlike canonical macroautophagy, LAP does not form a double-membrane autophagosome and is independent of the ULK1-FIP200 initiation complex, but it uses the ATG5-ATG12-ATG16L1 conjugation machinery to lipidate LC3 on the phagosomal membrane; Rubicon and NOX2-derived ROS may serve as characteristic regulators. Xenophagy, by contrast, captures cytosolic microbes or damaged pathogen-containing vacuoles within double-membrane autophagosomes.

LAP promotes phagosome maturation and lysosomal fusion, thereby supporting microbial killing, efferocytosis, and inflammatory resolution. Its dysregulation has been implicated in infectious, autoimmune, inflammatory, and neurodegenerative disease contexts. However, LC3 decoration alone cannot distinguish LAP from other non-canonical ATG8-lipidation pathways and does not establish successful lysosomal degradation; pathway identity and completion require assessment of membrane topology, pathway-specific regulators, maturation, and cargo turnover (22–24).

Autophagy contributes to inflammatory control by clearing pro-inflammatory organelles and signaling proteins, including RIG-I, IPS1/MAVS, inflammasome components, cytosolic DNA, and depolarized or damaged mitochondria.

Autophagy also regulates immune cell metabolism, as insufficient autophagy can drive naive immune cells toward glycolysis-dependent pro-inflammatory phenotypes rather than the oxidative metabolism required for regulatory T-cell and M2 macrophage survival (25). In neutrophils, autophagy displays distinct functional characteristics that support neutrophil-specific roles in immune defense and development (25). Key features include the regulation of neutrophil granulopoiesis in the bone marrow, the control of degranulation, and the generation of ROS. Impaired autophagy affects ROS generation, which in turn reduces degranulation, and consequently compromises the antimicrobial capacity of neutrophils (25). In addition to these functions, xenophagy, LAP, and the secretion of cytokines such as IL-1β are core autophagy-related functions in neutrophils (25).

Defective autophagy is linked to a wide range of pathological conditions, reflecting its diverse physiological roles. Monogenic defects in core autophagy genes illustrate the immunological relevance of this pathway, producing a clinical spectrum that prominently includes immunodeficiency, recurrent infections, and immune dysregulation (6). Vici syndrome is the prototypical congenital disorder of autophagy and is caused by biallelic variants in *EPG5*, which encodes a tethering factor required for correct autophagosome-lysosome fusion and endocytic trafficking (4, 26). The wide phenotypic spectrum of *EPG5*-related disorders likely reflects the functional impact of the specific variant on EPG5 protein function: Vici syndrome represents the more severe end of this spectrum, but probably not the most common clinical subgroup (3). Vici syndrome is defined by a combination of characteristic symptoms including agenesis of the corpus callosum, cataracts, cardiomyopathy, hypopigmentation, and combined immunodeficiency. Recurrent infections, in particular bacterial pneumonia and sepsis, are a leading cause of morbidity and mortality (H. 4, 5, 26). It has been demonstrated that EPG5 deficiency impairs TLR9 signaling by disrupting CpG DNA trafficking to the late endosomal-lysosomal compartment, a step essential for memory B-cell survival and plasma cell differentiation (6). The immunological profile in Vici syndrome ranges from near-normal to severe combined immunodeficiency with primarily humoral defects (hypogammaglobulinemia, impaired vaccine responses, memory B-cell loss) being most prominent. T-cell abnormalities have also been reported, as have hyperinflammatory states, including hemophagocytic lymphohistiocytosis (HLH), in some cases (27, 28). Recent work has implicated cGAS-STING-driven hyperinflammation as a neurodegenerative mechanism arising from mitochondrial-DNA mislocalization caused by defective mitophagy (7).

Beyond Vici syndrome, several additional monogenic autophagy and vesicular trafficking disorders feature (summarized in **Table 1**) prominent immune abnormalities (3). Chediak-Higashi syndrome, caused by biallelic variants in *LYST*, is characterized by impaired lysosomal trafficking, resulting in neutropenia, defective natural killer (NK) cell function, and recurrent pyogenic infections. Similarly, Griscelli syndrome type 2, caused by biallelic variants in *RAB27A*, is associated with impaired lytic granule exocytosis in cytotoxic T-cells and NK cells, predisposing to HLH. More recently, biallelic variants in *ATG9A* have been linked to severe Epstein-Barr virus (EBV)-driven lymphoproliferative disease, accompanied by defective NF-kB signaling and impaired autophagic activity.

**Table 1:** Overview of paradigmatic disorders affecting autophagy, endosomal trafficking, and lysosome-related organelles with immunodeficiency.

Disease	Disease gene	Cellular defect	Key immune phenotype	Clinical consequence
Vici syndrome	*EPG5*	Defective autolysosomal and/or amphisomal tethering	B cell immunodeficiency, cGAS-STING hyperinflammation	Life-threatening bacterial infections
Chediak-Higashi syndrome	*LYST*	Defective lysosomal trafficking; abnormal granule organization in NK cells and granulocytes	Congenital immunodeficiency with impaired NK cell function and neutropenia	Recurrent pyogenic infections, oculocutaneous hypopigmentation, lymphohistiocytosis
Griscelli syndrome type 2	*RAB27A*	Defective lytic granule exocytosis in cytotoxic T cells, NK cells, and melanocytes	Impaired T cell and NK cell degranulation with absent perforin-mediated cytotoxicity	Partial albinism, life-threatening recurrent HLH episodes
ATG9A deficiency	*ATG9A*	Impaired autophagosomal membrane expansion and autophagic activity; altered NF-κB signaling	Severe EBV-driven lymphoproliferative disease with hyperplastic T- and B-cell proliferations	Lymphoproliferation in lung and brain; defective autophagic activity in peripheral leukocytes
Hermansky-Pudlak syndrome	AP3-complex subunit genes, BLOC-genes	Defective biogenesis and trafficking of endosomes and lysosome-related organelles (melanosomes, platelet dense granules)	Subtype-dependent immunodeficiency featuring neutropenia, impaired NK cell function and defective cytotoxic T cell degranulation	Oculocutaneous hypopigmentation, bleeding diathesis, granulomatous colitis, progressive pulmonary fibrosis
Cohen syndrome	*VPS13B*	Golgi integrity disruption, endosomal trafficking defect	Intermittent or chronic neutropenia	Intellectual disability, obesity, myopia, retinal dystrophy

These disorders affect distinct cellular processes rather than a single xenophagy/autophagy continuum: LYST primarily alters lysosomal trafficking and granule organization, RAB27A regulates lytic-granule exocytosis, and ATG9A contributes to autophagosomal membrane expansion. Direct evidence that these lesions alter NET formation is limited; any such connection is therefore considered hypothesis generating.

The endosomal system serves as a central hub that coordinates sorting, recycling, and lysosomal degradation of endocytosed cargo while also regulating intracellular signaling in a spatiotemporal manner. Disorders of endosomal trafficking can present with neurodevelopmental abnormalities, hypopigmentation and/or immunodeficiency such as in AP-3 complex-related Hermansky-Pudlak syndrome (29, 30) or *VPS13B*-related Cohen syndrome (31). By organizing intracellular transport, endosomal machinery fine-tunes responses to external stimuli (32, 33–35) and maintains intra- and extracellular homeostasis (33, 34, 36). In innate immunity, endosomal transport serves as a critical checkpoint for immunological signaling (37–40). Endosomes are positioned at the interface between receptor signaling and degradation, and regulate the fate of immune receptors and ubiquitinated proteins by coupling signal transduction to cargo turnover, thereby determining the duration and intensity of immune stimuli (37). The spatiotemporal regulation of immune signals, in particular key signaling axes such as TLR signaling and the IL-1β/NF-κB pathway, assigns a key role to the endosomal transport machinery (38, 41, 42). When receptor trafficking is impaired, degradation is delayed, signaling persists, and inflammatory responses are prolonged. Autophagy also contributes to immune regulation, and is functionally interconnected with the endosomal system (37, 40, 43, 44).

Xenophagy highlights the dependence of antimicrobial defense on intact intracellular trafficking and degradative pathways (45–47) (**Figure 1**). Efficient xenophagic clearance requires not only pathogen recognition, but also productive cargo delivery, vesicular maturation and regulated autophagosome-lysosome fusion, processes to which TOM1 may contribute through endosomal sorting and autophagosome maturation (48). TOM1 supports endosomal sorting and autophagosome maturation, processes that may facilitate xenophagic completion. Evidence for a universal, direct requirement for TOM1 in xenophagy is limited; defective TOM1 function is therefore discussed as a plausible contributor to impaired cargo clearance and sustained inflammatory signaling rather than as an established cause of defective xenophagy (43, 44, 47–51).

**Figure 1:**
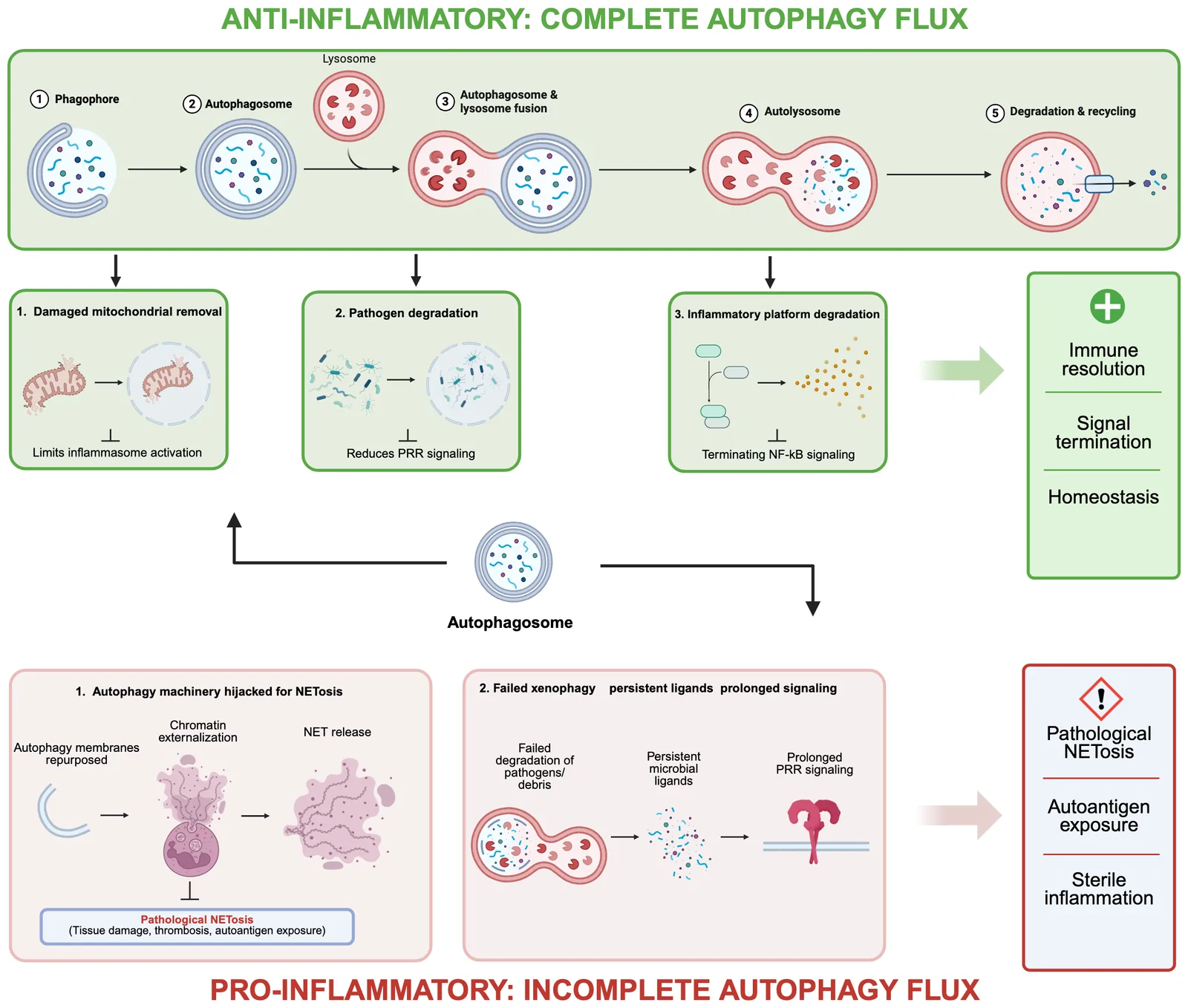
Autophagic flux as a checkpoint between immune resolution and pathological NET formation. Complete autophagic flux promotes immune resolution through sequential phagophore formation, cargo sequestration within autophagosomes, autophagosome–lysosome fusion, autolysosome formation, and lysosomal degradation and recycling. Selective removal of damaged mitochondria limits inflammasome activation, xenophagic degradation reduces the persistence of microbial ligands and pattern-recognition receptor (PRR) signaling, whilst degradation of inflammatory signaling platforms facilitates nuclear factor-κB (NF-κB) signal termination. These processes collectively restore cellular homeostasis. Conversely, autophagy can contribute to inflammation through two non-exclusive routes. First, early autophagic membranes and metabolic machinery may be repurposed under defined stimuli to support chromatin externalization and neutrophil extracellular trap (NET) release, with excessive NET formation promoting tissue injury, thrombosis, and autoantigen exposure. Second, failure of xenophagic degradation allows pathogens, cellular debris, and microbial ligands to persist, resulting in prolonged PRR signaling and sustained neutrophil priming. In this conceptual model, both routes could lower the threshold for pathological NET formation and perpetuate autoantigen exposure and sterile inflammation. The model emphasizes that the immunological consequences of autophagy depend on the affected pathway stage and whether autophagic flux proceeds to lysosomal degradation. The proposed NET-promoting links are stimulus dependent and should not be interpreted as a universal causal sequence; LC3 recruitment alone does not establish degradative flux. NET, neutrophil extracellular trap; NF-κB, nuclear factor-κB; PRR, pattern-recognition receptor. Created with BioRender.com.

The Target of Myb1 (TOM1) family comprises TOM1, TOM1-L1, and TOM1-L2. These proteins act as early adaptors associated with ESCRT-0 functions and link membrane trafficking, autophagy, and immune signaling (52). TOM1 is predominantly cytoplasmic and localizes to early endosomes, where its modular domain organization enables cargo recognition and sorting. Its GGA and TOM1 (GAT) domain binds polyubiquitinated proteins, while the Vps27, Hrs and STAM (VHS) domain mediates phosphatidylinositol-5-phosphate (PI5P) binding. In complex with Toll-interacting protein (TOLLIP) and endofin, TOM1 recruits ubiquitinated cargo to early endosomes, promotes endosomal maturation and clathrin-mediated sorting, thereby dampening TLR signaling. TOM1 also contributes to inflammatory resolution by facilitating immune receptor recycling (44, 53–58). TOM1 further connects endocytosis to autophagy through its C-terminal interaction with myosin VI, a protein that associates with LC3B on autophagosomes and couples endosomal transport to autophagic maturation. This pathway facilitates amphisome formation, autophagosome maturation, lysosomal fusion and efficient degradation of endosomal cargo. In addition, recruitment of autophagy receptors such as NDP52, optineurin, and TAX1BP1 expands the range of cargo targeted for autophagic degradation (48, 59).

TOM1 is a negative regulator of IL-1R1 signaling and contributes to timely inflammatory-signal termination (**Figure 2**). After IL-1β binding, IL-1R1 is endocytosed, and TOLLIP participates in trafficking ubiquitinated receptor complexes. TOM1 is recruited through the TOM1–TOLLIP interaction; its GAT domain mediates interactions with ubiquitin and TOLLIP, whereas its VHS domain contributes to PI5P-dependent endosomal association. Together, these interactions support sorting toward lysosomal degradation. Functional studies implicate the TOM1–TOLLIP axis in limiting IL-1β- and TNF-α-induced NF-κB and activator protein 1 (AP-1) activity, although the precise receptor-to-domain contacts remain incompletely resolved (43, 44, 55, 60). TOM1 prevents the accumulation of signaling-competent IL-1R1 in late endosomes by facilitating lysosomal receptor turnover, thus restricting both the magnitude and the duration of inflammatory signaling. Conversely, TOM1 deficiency impairs receptor degradation and sustains activation of the Myeloid differentiation primary response protein MyD88-dependent signaling cascade. This includes Interleukin-1 receptor-associated kinase 1, TNF receptor-associated factor 6, Mitogen-activated protein kinase kinase 7, and the IκB kinase complex, culminating in enhanced NF-κB activation and increased expression of pro-inflammatory mediators such as IL-6, IL-8, COX-2, and TNF-α (43, 44, 55, 60, 61). The physiological importance of this regulatory axis is underscored by its involvement in a wide range of human diseases, including neurodegenerative disorders. In Alzheimer’s disease (AD), *TOM1* expression is markedly reduced in the hippocampus both in patients and corresponding mouse models, associated with altered microglial activity, elevated amyloid-β (Aβ) levels, and cognitive decline (43, 62). Restoring *TOM1* expression alleviates these pathological features, highlighting the TOM1–IL-1R1 signaling pathway as a potential therapeutic target for neuroinflammatory and neurodegenerative diseases (43). A *TOM1* missense variant reported in a mother–son pair with a combined immunodeficiency–autoimmunity syndrome further supports a role for TOM1 in immune homeostasis. The phenotype in this family included recurrent respiratory infections, lymphocytic interstitial pneumonitis, progressive pulmonary fibrosis, atopic eczema, psoriasis vulgaris, chronic diarrhea, autoimmune enteropathy, seronegative polyarthritis, hypogammaglobulinemia, persistent low-copy EBV viremia, and growth impairment (61, 63). Collectively, these findings indicate a broader defect in immune homeostasis. A resulting change in NET-formation thresholds is biologically plausible but has not been directly demonstrated in this family.

**Figure 2:**
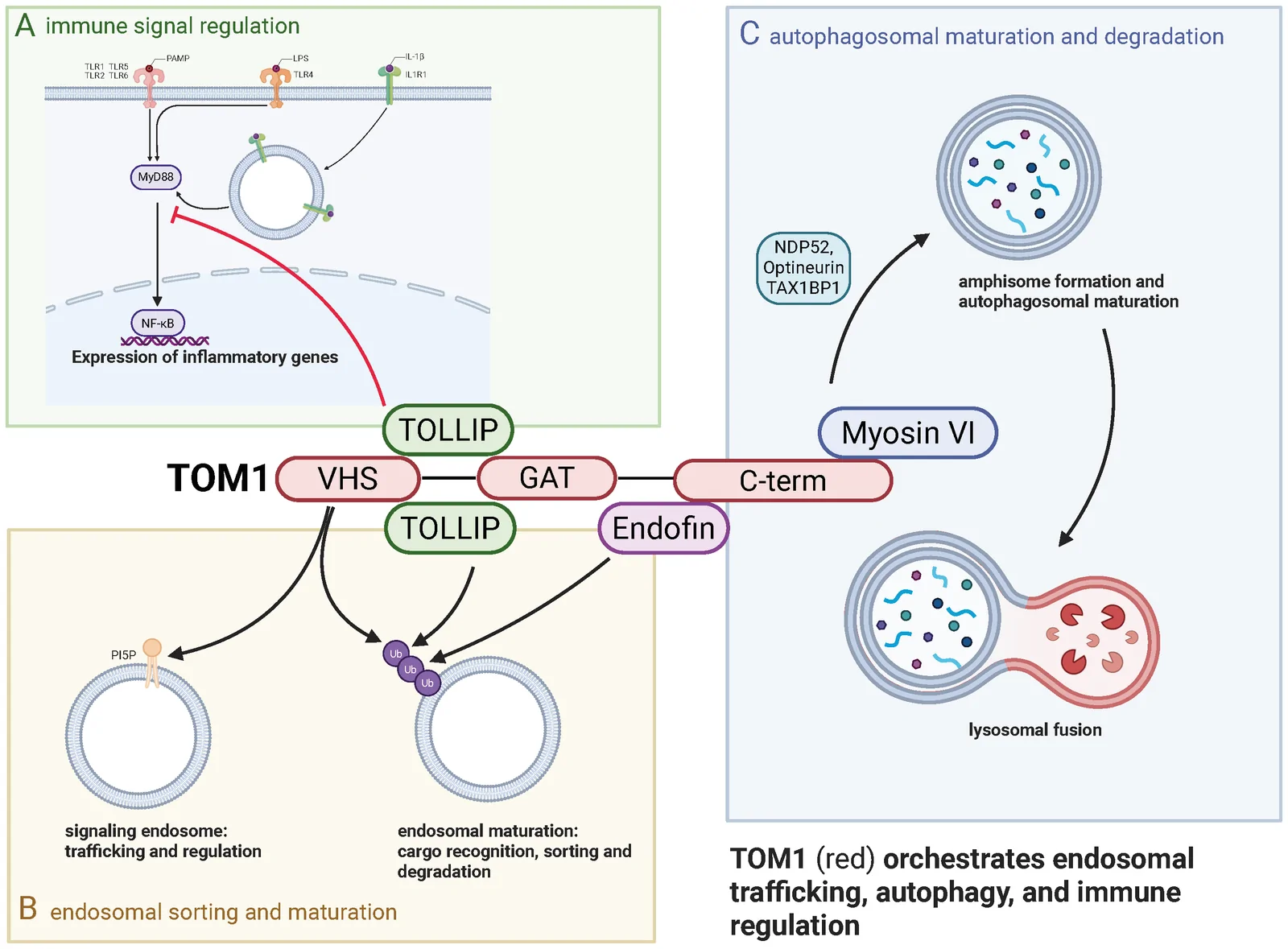
TOM1 coordinates endosomal trafficking, autophagosomal maturation, and innate immune regulation. **(A)** Immune-signal regulation. Activation of representative Toll-like receptors (TLRs) by pathogen-associated molecular patterns (PAMPs) or lipopolysaccharide (LPS), and of interleukin-1 receptor type 1 (IL-1R1) by interleukin-1β (IL-1β), induces MyD88-dependent activation of nuclear factor-κB (NF-κB) and inflammatory gene expression. Through its interaction with TOLLIP, TOM1 couples activated-receptor trafficking to endosomal sorting and lysosomal turnover, thereby contributing to inflammatory-signal termination. **(B)** Endosomal sorting and maturation. TOM1 comprises an N-terminal VHS domain, a central GAT domain, and a C-terminal region. PI5P-dependent membrane association recruits TOM1 to signaling endosomes, whereas the GAT domain mediates interactions with ubiquitin and TOLLIP. Together with TOLLIP and Endofin, TOM1 facilitates the recognition, sorting, and trafficking of ubiquitinated cargo toward endosomal maturation and degradation. **(C)** Autophagosomal maturation and degradation. Interaction of the TOM1 C-terminal region with myosin VI connects TOM1-positive endosomal membranes with autophagosomes. Docking of myosin VI to the autophagy receptors NDP52, optineurin, and TAX1BP1 facilitates endosomal cargo delivery, amphisome formation, autophagosomal maturation, and subsequent fusion with lysosomes. TOM1 therefore acts as an adaptor linking endosomal cargo sorting, autophagic degradation, and immune-signal resolution. GAT, GGA and TOM1 domain; MyD88, myeloid differentiation primary response 88; NDP52, nuclear dot protein 52 kDa; PI5P, phosphatidylinositol 5-phosphate; TAX1BP1, Tax1-binding protein 1; TOLLIP, Toll-interacting protein; TOM1, target of Myb1 membrane trafficking protein; Ub, ubiquitin; VHS, Vps27, Hrs, and STAM domain. Created with BioRender.com.

Beyond its role as a cargo adaptor, TOM1 also functions as a direct effector of PI5P, linking endosomal lipid composition to cargo fate. Binding of PI5P to the conserved VHS domain recruits TOM1 to signaling endosomes, delays endosomal maturation and thus prolongs receptor residence while limiting lysosomal degradation. Consequently, the PI5P–TOM1 axis fine-tunes the balance between sustained receptor signaling required for immune activation and timely signal termination essential for inflammatory resolution (44, 53, 64). This regulatory mechanism is further refined by pH-dependent changes in TOM1 function. Although the TOM1–TOLLIP complex remains stable in the acidic environment of late endosomes, TOM1 loses its ubiquitin-binding capacity. This pH-dependent functional switch establishes a compartment-specific control point that ensures site-specific fine-tuning of cargo-sorting decisions. Together, the PI5P–TOM1 axis acts as a direct regulatory switch at the interface of endosomal membrane dynamics, cargo sorting, and immune regulation (65).

Endosomes function not only as passive transport intermediates, but as dynamic regulatory hubs that govern the magnitude, duration, and resolution of innate immune signaling. By integrating receptor trafficking with cargo sorting and degradation, they establish checkpoints that balance sustained immune activation with timely signal termination, thus ensuring inflammatory resolution (32, 37, 39, 66). These mechanisms extend the concept of immune resolution beyond receptor downregulation and highlight its direct immunopathological relevance. Pathogenic *TOM1* variants illustrate this principle, causing severe early-onset multi-organ autoimmunity associated with combined immunodeficiency (44, 48, 55, 61, 63). Defective endosomal trafficking has emerged as a common feature of autoimmune, chronic inflammatory, neuroinflammatory and immunodeficiency disorders, underscoring the importance of endosomal checkpoints in determining receptor fate and inflammatory outcome. Persistent inflammation can therefore arise not only from excessive immune activation, but also from defective termination of receptor signaling (32, 43, 44, 53, 61–63).

EPG5 and TOM1 are both functionally linked to endosomal–lysosomal homeostasis and have been associated with overlapping neuroimmunological phenotypes (4, 43, 61, 63). Mechanistically, the two proteins occupy distinct hierarchical positions within the vesicular trafficking system. EPG5 functions as a core component of the autophagic machinery, directly controlling autophagosome–lysosome fusion and autophagic flux, whereas TOM1 acts as a modulatory adaptor within endosomal sorting pathways, fine-tuning intracellular trafficking efficiency and signal resolution. This distinction suggests that the clinically convergent phenotypes may arise from perturbations at different levels of the endolysosomal system, reflecting either impaired execution of autophagy or dysregulated control of endosomal trafficking dynamics (4, 26, 43, 48, 61, 63). This framework extends the concept of endosomal checkpoint regulation beyond inflammatory receptor signaling and suggests a potential, but not directly demonstrated, route by which membrane trafficking could influence neutrophil activation and NET-associated immunopathology (9, 43, 48, 53, 67–69).

## 4 The autophagy-NETs network: molecular mechanisms

The relationship between autophagy-related processes and NET formation is context dependent rather than a single coordinated program. Studies using selected stimuli have associated early autophagy machinery, metabolic shifts, or ROS production with lytic NET release, whereas other models show different dependencies. Here, the term autophagic flux is reserved for demonstrated progression to lysosomal degradation; LC3 recruitment or autophagosome accumulation is described separately (9). In PMA and other ROS-dependent experimental systems, superoxide generation and autophagy-related signaling have been reported to cooperate during lytic NET release; these findings should not be generalized to all NET-inducing stimuli or to vital NET formation (9). Autophagy-related membrane and metabolic responses may therefore facilitate chromatin externalization in defined settings, while completed degradative flux can reduce inflammatory triggers through a separate mechanism.

The molecular regulation of NET formation by the autophagic machinery involves a complex interplay between nutrient-sensing pathways, oxidative stress, and selective cargo adaptors. Combined autophagy-related signaling and superoxide generation has been reported to facilitate lytic NETosis in selected models, but neither dependency is universal across stimuli (9).

The mTOR/Akt pathway is one stimulus-sensitive regulator of the autophagy-NET relationship rather than a universal master switch (9, 20). The mTOR/Akt signaling pathway negatively regulates autophagy to maintain cellular homeostasis under basal conditions. Under selected conditions, TLR ligands, hypoxia, or pro-inflammatory cytokines can inhibit mTOR activity and relieve suppression of autophagy-related pathways (4). This does not by itself establish restoration of degradative flux. Pharmacological mTOR blockade has promoted NET formation in some neutrophil models, potentially through early autophagy-related and metabolic effects. Inflammatory stimuli such as lipopolysaccharide can induce autophagy under physiological conditions, supporting the concept that autophagic signaling may modulate NET formation in a context-dependent manner, with variable effects reported across stimuli and experimental systems (11, 70).

Autophagy in neutrophils is closely linked to ROS production (25): NADPH oxidase-dependent superoxide generation is important for the initial phases of many lytic NET models (10). Selective autophagy receptors such as p62/SQSTM1 and TAX1BP1 further modulate this response by regulating the degradation of pro-inflammatory adaptors like TRIF, which acts downstream of TLR3 and TLR4, and may thus limit the magnitude of inflammatory signaling that can prime NET formation (20). Overall, the effect of autophagy-related pathways on NET release depends on stimulus, experimental definition, and whether initiation, autophagosome accumulation, or degradative completion is measured.

Peptidylarginine deiminase 4-mediated citrullination provides one of the best-characterized molecular bridges between neutrophil activation and the exposure of modified self-antigens. PAD4 catalyzes the conversion of arginine residues to citrulline on histones and other proteins, thus reducing electrostatic interactions between histones and DNA, promoting chromatin decondensation, and ultimately facilitating the nuclear remodeling required for NET release. This step may be particularly relevant to autoimmunity, because citrullinated histones and other citrullinated proteins can serve as autoantigens, especially in rheumatoid arthritis and lupus-like inflammatory settings. However, PAD4 is not ubiquitously required for all forms of NET formation. PAD4-dependent and PAD4-independent programs coexist, and their relative contribution varies depending on stimulus, tissue context, species, and the distinction between vital NET release and lytic NETosis.

A central pathogenic feature of NET biology is the ability to amplify inflammation through feed-forward mechanisms. NET-derived DNA, histones, neutrophil elastase, myeloperoxidase and citrullinated proteins can activate plasmacytoid dendritic cells, macrophages, endothelial cells and neighboring neutrophils. NET-associated nucleic acids may promote type I interferon and inflammasome-associated cytokine programs, whereas histones and proteases directly damage tissues and endothelial surfaces. This can create a self-reinforcing circuit in which NETs not only reflect neutrophil activation, but also amplify the cytokine and DAMP-rich inflammatory environment that drives further NET formation. This loop may be particularly harmful in autoimmunity when NETs combine structural autoantigen exposure with innate adjuvanticity. Defective DNase-mediated degradation or inefficient macrophage clearance can further prolong NET persistence and thereby convert a physiological antimicrobial program into chronic sterile inflammation.

## 5 Autophagy-NETs dysregulation in autoimmune disease

The autophagy-NETs network provides a unifying framework for understanding why autoimmune diseases frequently combine excessive innate immune activation with defective clearance of self-derived material (71).

In systemic lupus erythematosus, NETs expose dsDNA, histones, LL37, myeloperoxidase and other immunostimulatory molecules to immune complexes and nucleic-acid sensors. In rheumatoid arthritis, citrullinated proteins and neutrophil-derived enzymes contribute to a microenvironment in which NET formation can reinforce autoantibody production and synovial inflammation. In ANCA-associated vasculitis, primed neutrophils release NET components that can further present MPO and proteinase-3 as autoantigens, linking neutrophil effector function to vascular injury. NETs are not merely products of inflammation across these diseases, but active platforms that can sustain autoreactivity. **Table 2** demonstrates several key autoimmune diseases in this context.

**Table 2:** Overview of autoimmune diseases with autophagy-NETs dysregulation.

Disease	Key NET components exposed	Central NET-driven mechanism	Pathological consequence
Systemic lupus erythematosus (SLE)	dsDNA histones, LL37, MPO, tissue factor, IL-17A	NETs serve as source of extracellular autoantigens. Immune complexes with NET components activate dendritic cells via nucleic-acid sensors, driving type I IFN production. NETs promote thromboinflammation via RF-bearing and IL-17A-bearing structures in target tissues.	Nephritis, skin lesions, vascular damage; excess NET burden reflects increased formation and/or impaired clearance; any autophagy-related contribution is context dependent
Rheumatoid arthritis (RA)	Citrullinated histones, citrullinated fibrinogen, NE, MMP8	Citrullinated proteins generated via PAD2/4 activity are recognized by ACPAs, forming immune complexes that drive further NET formation; NETs engulfed by fibroblasts stimulate anti-citrullinated antibody production	Synovial inflammation, cartilage and bone erosion; NETs can reinforce a self-perpetuating loop between NET formation and autoantibody production
ANCA-associated vasculitis (AAV)	MPO, PR3, LAMP-2	ANCAs (targeting MPO and PR3) prime neutrophils and enhance NET release; anti-LAMP-2 autoantibodies have been reported to stimulate NET formation through autophagy-related signaling, but the generalizability of this mechanism remains uncertain	Neutrophil-mediated vascular injury; NET components presented as autoantigens further sustain the autoimmune response

Autophagy and xenophagy influence this process at multiple stages: Upstream, effective xenophagic clearance reduces the intracellular burden of pathogens or other danger-associated material that drives neutrophil activation. Downstream, efficient endolysosomal processing and macrophage-mediated clearance facilitate the removal of extracellular debris after immune activation. Inflammation is prolonged through multiple interconnected mechanisms when these systems fail. Consequently, microbial or sterile triggers may persist, damaged organelles may continue to generate ROS, cytosolic nucleic acids may remain available for sensing, and extracellular NET material may accumulate. These observations help explain why molecular families that support pathogen clearance can also preserve immune tolerance. The literature on autophagy-related (ultra-)rare diseases is particularly informative here, as it provides human genetic models showing that distinct defects in trafficking or autophagosome-lysosome fusion can manifest as combined immunodeficiency, infection susceptibility, autoimmunity, and hyperinflammation without implying a single shared xenophagy mechanism (3, 4).

Pathogenic *TOM1* variants and reduced TOM1 activity provide a mechanistic model in which impaired receptor degradation and defective autophagosome-lysosome fusion may converge on NF-κB activation, cytokine amplification, and immune dysregulation. Whether this changes NET-formation thresholds in neutrophils remains to be tested, especially in regards to altered NET priming as a testable, indirect consequence of persistent inflammatory stimuli rather than as an established TOM1 phenotype.

## 6 Barrier-centered immune dysregulation and failed resolution in allergic diseases

Allergic diseases are immunologically distinct from autoimmune disorders, although both can involve persistent inflammation and failed resolution. Allergic diseases have traditionally been conceptualized predominantly through the lens of type 2 immunity (72, 73). This framework does not fully capture their biological complexity. Many allergic diseases arise at epithelial barrier surfaces, including the skin, respiratory tract and gastrointestinal mucosa, where epithelial integrity constitutes the first line of defense against environmental allergens, microbes and pollutants (74). Persistent epithelial stress and defective barrier repair continuously expose the immune system to environmental allergens, microbial products and damage-associated molecular patterns (DAMPs), thereby sustaining innate immune activation and establishing a tissue microenvironment that favors chronic inflammation over restoration of tissue homeostasis (73). Persistent barrier dysfunction also promotes reciprocal interactions between structural cells, resident immune cells and infiltrating granulocytes, generating self-perpetuating inflammatory circuits that contribute to chronic tissue remodeling and disease persistence (72).

Within this framework, autophagy emerges as a central regulator of epithelial homeostasis, microbial clearance and inflammatory resolution. Beyond its canonical role in intracellular degradation, autophagy contributes to epithelial barrier integrity by maintaining mitochondrial quality control, limiting oxidative stress, regulating inflammasome activation and facilitating the removal of damaged proteins, intracellular pathogens and dysfunctional organelles. These functions are increasingly recognized as essential for preserving epithelial resilience under conditions of chronic environmental exposure and inflammatory stress (19). Autophagy also modulates antigen processing, cytokine production and innate immune signaling through interactions with endolysosomal trafficking pathways, thereby influencing both epithelial and immune cell responses. Consequently, impaired autophagic flux may compromise epithelial barrier integrity, alter host–microbiota interactions, amplify oxidative stress and prolong inflammatory signaling, establishing conditions that favor chronic barrier inflammation, defective tissue repair and persistent activation of innate immune pathways rather than restoration of tissue homeostasis (19). Because autophagy regulates stress adaptation and inflammatory signaling in both structural and immune cells, it may represent a common upstream regulator linking epithelial homeostasis with inflammatory resolution across multiple allergic diseases. From this perspective, maintenance of epithelial barrier integrity can itself be regarded as an active component of immune resolution rather than merely a passive physical defense.

Among allergic diseases, the relationship between autophagy and chronic barrier inflammation has been investigated most extensively in asthma, where accumulating evidence links altered autophagic activity to epithelial dysfunction, oxidative stress, airway remodeling and chronic inflammation (75). While asthma has traditionally been regarded as a predominantly type 2-driven inflammatory disease characterized by eosinophilic airway inflammation mediated by IL-4, IL-5 and IL-13, increasing recognition of distinct inflammatory endotypes has challenged this paradigm (72). Whereas type 2 (T2)-high asthma is characterized by eosinophilic inflammation and generally responds well to corticosteroids and biologic therapies targeting IgE, IL-5 or IL-4/IL-13 signaling, T2-low asthma frequently exhibits neutrophilic or paucigranulocytic inflammation, reduced corticosteroid responsiveness and a less well-defined molecular pathogenesis (76). Although less common than T2-high disease, T2-low endotypes are overrepresented among patients with severe, treatment-refractory asthma and remain a major unmet therapeutic challenge. Persistent epithelial injury may continuously release endogenous DAMPs while facilitating repeated exposure to inhaled environmental stimuli, thereby maintaining innate immune activation despite the absence of persistent invasive infection. Whether impaired autophagic flux contributes to defective inflammatory resolution in T2-low asthma remains unknown. However, given the central role of autophagy in epithelial homeostasis, innate immune regulation and the active termination of inflammation, such a mechanism represents an attractive concept that deserves further investigation. This model may help explain why conventional anti-inflammatory strategies including corticosteroids often incompletely suppress disease activity despite reducing inflammatory cytokine production. Corticosteroids may inhibit inflammatory gene transcription, whereas they are unlikely to fully restore the endogenous mechanisms responsible for termination of inflammation and repair of epithelial barrier integrity.

Within this framework, neutrophils may assume a more central role in T2-low asthma than traditionally appreciated. Persistent activation of NET-associated effector pathways may shift protective innate immune responses towards chronic epithelial injury and defective tissue repair, thereby reinforcing airway remodeling and perpetuating inflammation despite the absence of uncontrolled infection. Rather than merely reflecting exaggerated inflammation, persistent neutrophilic airway inflammation may indicate failure of endogenous resolution mechanisms, potentially linking impaired autophagic function, defective endolysosomal trafficking and chronic tissue damage. Thus, defective intracellular clearance and insufficient inflammatory resolution may shift protective innate immune responses towards persistent tissue-damaging inflammation.

Regarding T2-high asthma, eosinophils, which represent the principal granulocyte population in T2-high disease, are likewise capable of releasing extracellular traps (EETs), which contribute to antimicrobial host defense but have also been implicated in airway inflammation, mucus plugging and tissue injury in eosinophilic airway diseases (77). Hence, dysregulated extracellular trap formation may represent a shared innate immune mechanism through which persistent danger signaling contributes to chronic inflammation across both autoimmune and allergic diseases, albeit involving different dominant granulocyte populations depending on the underlying inflammatory context. Taken together, these observations raise the possibility that, in at least a subset of patients, severe T2-low asthma may reflect defective inflammatory resolution rather than merely quantitatively greater inflammation. In this context, persistent epithelial injury, sustained innate immune activation and impaired restoration of tissue homeostasis may collectively drive chronic airway inflammation despite the absence of persistent invasive infection. Identification of patients in whom defective epithelial homeostasis, impaired autophagic flux or dysregulated innate immune activation constitute dominant pathogenic mechanisms may therefore enable more individualized therapeutic approaches beyond conventional type 2-directed therapies.

The consequences of primary epidermal barrier failure are particularly evident in severe dermatitis, multiple allergies and metabolic wasting (SAM) syndrome. Biallelic loss-of-function variants in DSG1 or DSP impair desmosomal integrity and cause superficial epidermal acantholysis, skin fragility and early-onset dermatitis, frequently accompanied by multiple allergies and profound type 2 immune activation (78). Rather than simply illustrating the consequences of structural barrier disruption, SAM syndrome may serve as a human genetic model demonstrating how persistent barrier failure can initiate and sustain chronic allergic inflammation. Continuous epithelial injury may provide a persistent source of endogenous danger signals that perpetuate innate immune activation while preventing restoration of tissue homeostasis. Because the underlying structural defect cannot be repaired by anti-inflammatory therapy, restoration of tissue homeostasis may depend even more strongly on endogenous mechanisms that terminate inflammation and limit epithelial stress. Although direct evidence is currently lacking, persistent epithelial injury may ultimately overwhelm endogenous mechanisms of inflammatory resolution. In this context, dysregulated autophagy, defective endolysosomal signaling and persistent extracellular trap formation represent plausible candidate mechanisms that merit future investigation. If validated, this concept would imply that both SAM syndrome and severe, treatment-resistant atopic dermatitis are characterized not only by profound barrier dysfunction and type 2 inflammation but also by impaired restoration of tissue homeostasis, whereby failed inflammatory resolution may contribute to disease persistence and therapeutic refractoriness. Taken together, these observations support a broader conceptual framework in which chronic allergic disorders are viewed not solely as disorders of exaggerated type 2 immunity but as diseases of persistent epithelial danger signaling and defective restoration of tissue homeostasis (19, 75). Across the skin, respiratory tract and gastrointestinal mucosa, epithelial barrier disruption may continuously expose the immune system to environmental allergens, microbes and DAMPs, thereby maintaining activation of both innate and adaptive immune pathways. Within this model, chronic inflammation reflects not only excessive immune activation but also failure to adequately resolve epithelial injury and restore tissue homeostasis. Autophagy may represent a central intracellular component of this resolution program by promoting cellular quality control, antimicrobial defense and termination of inflammatory signaling, whereas impaired autophagic flux could favor persistent innate immune activation and chronic tissue damage. In parallel, dysregulated formation or impaired clearance of neutrophil and eosinophil extracellular traps may further amplify innate inflammatory circuits and delay restoration of epithelial homeostasis.

## 7 Therapeutic implications

Therapeutic strategies targeting the autophagy-NETs network must account for the stage-specific and context-dependent effects of autophagy. Uniform stimulation or inhibition of autophagy is unlikely to be sufficient in many clinical settings. Enhancing degradative completion may improve xenophagic pathogen clearance, reduce cytosolic cargo, and shorten the duration of inflammatory signaling. By contrast, stimulating early autophagy without improving autophagosome-lysosome fusion and degradative completion may increase autophagosome accumulation or facilitate the membrane-remodeling requirements of selected forms of lytic NET release without resolving inflammatory cargo. Similarly, inhibiting autophagy may reduce NET formation in selected experimental settings but could simultaneously impair intracellular pathogen clearance and increase sterile danger-associated material accumulation.

Downstream anti-NET approaches include DNase-based strategies to degrade extracellular DNA scaffolds, PAD4 inhibition to reduce histone citrullination and chromatin decondensation, and modulation of ROS-producing pathways. These approaches may be particularly useful when NET burden itself is the predominant driver of tissue injury, as in selected forms of lupus nephritis, vasculitis, immunothrombosis or sepsis-associated thromboinflammation. However, such approaches do not necessarily correct upstream defects in xenophagic clearance, endosomal signal termination or lysosomal flux. Combination strategies may therefore be required to simultaneously reduce pathologic extracellular NET persistence and restore intracellular immune resolution.

The lupus peptide P140/rigerimod may be particularly relevant for the selective modulation of lysosomal and autophagic antigen processing in autoimmunity (79). P140 has been proposed to interfere with HSC70-dependent lysosomal pathways and endogenous MHC class II antigen presentation, thus recalibrating autoreactive immune activation without broad cytotoxic immunosuppression (80–82). This work is of both conceptual and therapeutic relevance, as it provides evidence that selective targeting of the autophagy-antigen presentation interface may represent a therapeutic strategy for systemic autoimmune diseases. Future approaches may similarly aim to restore autophagosome-lysosome fusion, reinforce TOM1/TOLLIP-dependent receptor degradation, improve NET clearance, or selectively modulate neutrophil metabolic checkpoints according to disease state and infection risk (83).

## 8 Discussion and conclusion

The autophagy-NET framework should be viewed as a set of interacting, non-equivalent processes rather than as a linear sequence from autophagy to NET release. The relevant pathways include canonical autophagy, xenophagy, LAP, endosomal trafficking, autophagosome-lysosome fusion, and lysosome-related organelle secretion. They are mechanistically distinct even where they share proteins or converge on lysosomal handling. Their effects on receptor signaling, cargo persistence, and inflammatory priming may influence NET release, but NET formation is one possible extracellular effector outcome rather than an obligatory endpoint.

This perspective helps resolve apparent inconsistencies in the literature on autophagy and NET formation. Autophagy can be anti-inflammatory when completion of autophagic flux removes damaged mitochondria, limits inflammasome activation and cytosolic DNA release, and degrades intracellular pathogens and inflammatory signaling platforms. Early autophagy-related membrane and metabolic programs may also support chromatin externalization under selected stimuli. The divergent findings reflect genuine biological and methodological heterogeneity, including stimulus, species, cell state, NET definition, and whether initiation or degradative completion is measured. The relevant question is therefore which pathway stage is affected and whether cargo proceeds to effective lysosomal degradation.

Xenophagy may influence pathogen persistence and NET-formation thresholds. If intracellular microbes are efficiently ubiquitinated, recognized by selective receptors, and routed to lysosomes for degradation, the burden of inflammatory stimulus decreases. Failure of xenophagy allows microbial ligands, damaged endomembrane structures, ROS-generating organelles and PRR signals to persist, keeping neutrophils primed while surrounding macrophages, dendritic cells, and endothelial cells continue to produce cytokines that may lower the threshold for NET release. NET formation could then serve as both a compensatory antimicrobial response and a mechanism of sterile tissue injury, a duality that may be particularly relevant in patients with autophagy- or trafficking-related disorders (3, 5).

Endosomal checkpoints provide the mechanistic bridge because endosomes act as signaling compartments that determine whether receptors continue to signal or are targeted for degradation. TOM1 functionally links receptor sorting, autophagosome maturation, and inflammatory signal termination through its interactions with TOLLIP, ubiquitinated cargo, phosphoinositides and myosin VI-linked autophagy receptors. TOM1 is not a canonical effector of NET formation. Based on its roles in receptor sorting, autophagosome maturation, and inflammatory-signal termination, we propose that impaired TOM1 function could prolong IL-1R1/NF-κB signaling, compromise autophagosome-lysosome fusion, and create a cytokine environment conducive to pathological NET formation; this indirect link remains to be tested.

These principles can be illustrated by human monogenic disorders affecting lysosome-related organelles, endosomal trafficking, or autophagosome-lysosome fusion, without implying a shared proximal mechanism. Hermansky-Pudlak syndrome, particularly its AP-3-related forms, illustrates that disturbed biogenesis and trafficking of lysosome-related organelles can combine albinism and platelet dysfunction with neutropenia, immune defects and recurrent infections. Chediak-Higashi syndrome provides an even more direct example of defective lysosomal trafficking in neutrophils and cytotoxic lymphocytes, characterized by giant leukocyte granules, impaired antimicrobial function and a high risk of HLH. Griscelli syndrome type 2, caused by RAB27A deficiency, shifts the emphasis from degradative lysosomes to regulated cytotoxic granule exocytosis, further illustrating how a vesicular trafficking defect can give rise to both susceptibility to infection and hyperinflammation within the same clinical phenotype. LAMTOR2/p14 deficiency extends this principle to late endosome positioning and MAPK-dependent immune-cell signaling, leading to neutropenia, defective neutrophil-mediated bacterial elimination, impaired cytotoxic T-cell function, hypopigmentation, and recurrent infections. These syndromes are informative human models of distinct endosomal, lysosomal, or secretory defects. Whether they directly alter NET formation remains insufficiently studied.

Vici syndrome occupies a distinct but complementary position because the primary defect lies in EPG5-dependent autophagosome–lysosome fusion rather than in lysosome-related organelle secretion. Recurrent infections, humoral immune defects, impaired nucleic-acid trafficking and hyperinflammatory manifestations in *EPG5*-related disorders support the concept that completion of autophagic flux is critical for the effective termination of immune signaling. Vici syndrome thus links autophagy-related disorders with disorders of lysosome-related organelles. Across these conditions, microbial handling, immune effector function, and inflammatory resolution depend on intact vesicular maturation, fusion, or secretion through distinct proximal mechanisms. Direct evidence that these monogenic defects alter NET formation is limited. We therefore present altered NET priming or persistence as a testable hypothesis, while the pathogenic potential of insufficiently cleared extracellular chromatin is supported more broadly.

In autoimmunity, the decisive consequence is the persistent exposure of self-derived material with autoantigenic and pro-inflammatory potential (84). NETs present DNA, histones, myeloperoxidase, neutrophil elastase, LL37 and citrullinated proteins in a configuration that is both antigenic and pro-inflammatory, and as such capable of activating plasmacytoid dendritic cells, macrophages and B cells as well as reinforcing type I interferon, IL-1, IL-6, and TNF-rich inflammatory circuits. NETs can become persistent extracellular depots of autoantigens and danger signals when DNase-mediated degradation or phagocytic clearance is insufficient. The autophagy–NETs network therefore links impaired intracellular resolution to defective extracellular clearance, thus establishing a feed-forward system highly relevant for SLE, rheumatoid arthritis, ANCA-associated vasculitis and thromboinflammatory disease.

Therapeutically, this model argues against indiscriminate pathway modulation. Blocking PAD4, ROS, or NET scaffolds may be appropriate when extracellular NET burden drives tissue injury, but may not correct the upstream trafficking defect. Conversely, stimulating autophagy may only benefit patients if it enhances the completion of autophagic flux and xenophagic clearance, rather than merely increasing autophagosome initiation. The most promising interventions may therefore restore immune resolution particularly by improving autophagosome–lysosome fusion, enhancing endosomal receptor degradation, promoting macrophage-mediated NET clearance, and selectively controlling neutrophil metabolic activation. The work on P140/rigerimod is important here, as it provides proof of principle that lysosomal-autophagic antigen handling can be targeted with immunological specificity in lupus (79–82).

A major future challenge will be context-specific patient stratification. Autophagy-linked modulation of NET formation is unlikely to have the same pathogenic or therapeutic significance across acute infection, chronic sterile inflammation, monogenic immune dysregulation, lupus nephritis or thromboinflammatory sepsis. Biomarkers will need to distinguish autophagy initiation from completion of autophagic flux, intracellular pathogen persistence from sterile organelle damage, and NET production from NET clearance failure. These distinctions are essential because therapeutic inhibition of NET formation during infection may impair antimicrobial host defense, whereas insufficient NET clearance in autoimmunity may perpetuate tissue damage. A systems-level view of this network should therefore integrate neutrophil state, endosomal trafficking, lysosomal competence, cytokine environment and extracellular clearance capacity.

Taken together, canonical autophagy, xenophagy, LAP, endosomal trafficking, lysosome-related organelle secretion, and NET formation are related but non-equivalent processes at the boundary between defense and damage. Completed degradative flux and effective endosomal sorting can reduce intracellular danger and shorten inflammatory signaling, whereas defects in these pathways may sustain neutrophil priming. NETs can then amplify autoimmunity when formation is excessive or extracellular clearance is inadequate. We therefore offer the autophagy-NET framework as a testable conceptual model rather than a universal causal sequence, with restoration of intracellular resolution considered alongside control of extracellular inflammation.
